# Literature review of the burden of prostate cancer in Germany, France, the United Kingdom and Canada

**DOI:** 10.1186/s12894-019-0448-6

**Published:** 2019-03-18

**Authors:** J. Smith-Palmer, C. Takizawa, W. Valentine

**Affiliations:** 1 Ossian Health Economics and Communications GmbH, Bäumleingasse 20, 4051 Basel, Switzerland; 2 Genomic Health International, Geneva, Switzerland

**Keywords:** Prostate cancer, Cost of illness, France, Germany, United Kingdom, Canada

## Abstract

**Background:**

Prostate cancer is the most frequently reported cancer in males in Europe, and is associated with substantial morbidity and mortality. The aim of the current review was to characterize the clinical, economic and humanistic burden of disease associated with prostate cancer in France, Germany, the UK and Canada.

**Methods:**

Literature searches were conducted using the PubMed, EMBASE and Cochrane Library databases to identify studies reporting incidence and/or mortality rates, costs and health state utilities associated with prostate cancer in the settings of interest. For inclusion, studies were required to be published in English in full-text form from 2006 onwards.

**Results:**

Incidence studies showed that in all settings the incidence of prostate cancer has increased substantially over the past two decades, driven in part by increased uptake of prostate specific antigen (PSA) screening leading to earlier identification of tumors, but which has also led to over-treatment, compounding the economic burden of disease. Mortality rates have declined over the same time frame, driven by earlier detection and improvements in treatment. Both prostate cancer itself, as well as treatment and treatment-related complications, are associated with reduced quality of life.

**Conclusions:**

Prostate cancer is associated with a significant clinical and economic burden, whilst earlier detection and aggressive treatment is associated with improved survival, over-treatment of men with indolent tumors compounds the already significant burden of disease and treatment can lead to long-term side effects including impotence and impaired urinary and/or bowel function. There is currently an unmet clinical need for diagnostic and/or prognostic tools that facilitate personalized prostate cancer treatment, and potentially reduce the clinical, economic and humanistic burden of invasive cancer treatment.

**Electronic supplementary material:**

The online version of this article (10.1186/s12894-019-0448-6) contains supplementary material, which is available to authorized users.

## Background

In Europe, prostate cancer is now the most common cancer in men, accounting for 23% of all male cancers (*n* = 417,000 cases) and 10% of cancer-related deaths in males (*n* = 92,000 deaths) in 2012 [1]. Prostate cancers are heterogeneous in terms of presentation and morphology but nearly all are classified as adenocarcinomas and in many instances remain asymptomatic until locally advanced or metastatic. The incidence of prostate cancer is strongly related to age, and in the UK it is estimated that 1 in 8 men will be diagnosed with prostate cancer during their lifetime [2]. However, in a large proportion of men, prostate cancer is indolent or slow growing so may not become clinically significant during the lifetime of the patient, such that the number of men who die with prostate cancer by far exceeds the number of men who die due to prostate cancer. Indeed, autopsy studies in men who died from causes other than prostate cancer report an incidental prostate cancer prevalence at the time of death ranging from 5% at age < 30 years to 59% at age > 79 years [3].

In Western Europe and North America opportunistic screening for elevated prostate specific antigen (PSA) levels is commonly performed in middle-aged and elderly men. PSA is a serine protease that is expressed almost exclusively in the epithelial cells of the prostate gland and elevated PSA levels are a key indicator of prostate cancer. However, increased PSA levels can also occur as a result of several other conditions including, most notably, benign prostatic hyperplasia and prostatitis. Men with elevated PSA levels are therefore commonly referred for digital rectal examination (DRE) and/or biopsy to confirm the presence of cancer. In many settings PSA screening has contributed to enhanced detection, diagnosis and treatment of prostate cancer, but it is a contentious issue owing to the potential for over-diagnosis and over-treatment of prostate cancers that may not become clinically significant during the lifetime of the patient. Screening remains opportunistic rather than routine in many settings. In the United States, in 2008 the Preventative Services Task Force (USPSTF) recommended against routine screening due to its psychological harms and uncertainty around its clinical benefits [4]. However, the USPSTF recently updated its recommendations for men aged 55–69 years, stating that men should be informed of the potential benefits and harms of PSA screening and that the final decision should rest with the individual [5]. Other investigators have also suggested that population-based screening could lead to over-treatment [6].

In addition to the psychological harms cited by the US Preventative Services Task Force over-treatment may place an unnecessary burden on already overstretched health systems, and on an individual patient level increases the potential for treatment-related side-effects including impotence, impaired bowel function and incontinence, which can have a significant detrimental impact on quality of life. In particular, radical prostatectomy, the use of which has increased substantially in recent years, is generally advocated in men with localized disease with life expectancy > 10 years and who are willing to accept the risk of complications. Radical prostatectomy is associated with high medical resource use with reported median length of stay ranging from two to eight days in studies from the US and Europe [7–9]. A high proportion of men who undergo radical prostatectomy experience post-operative complications including erectile dysfunction and urinary incontinence, which can lead to impaired quality of life in terms of sexual and urinary function [10]. There is also a risk of complications for men who undergo radiation treatments, which can include painful urination, urinary incontinence, urethral stricture, rectal bleeding and/or leaking, erectile dysfunction and lymphedema. Moreover, such symptoms can negatively impact quality of life not only in men undergoing radical prostatectomy, but also in the partners of patients, who may also act as informal care givers in many instances.

The phenomenon of over-treatment is becoming increasingly recognized by physicians and consequently the strategies of watchful waiting and active surveillance are now recommended in several guidelines for men with low risk/early stage prostate cancer [11, 12]. Moreover, an increased uptake in active surveillance in recent years likely reflects efforts to reduce the over-treatment of low risk prostate cancer. The recently published ProtecT trial compared 10-year outcomes with active monitoring compared with surgery or radiotherapy in men with localized prostate cancer detected during PSA testing. Overall, 10-year prostate cancer-specific mortality rates were low in all trial arms and not significantly different between arms. However, significantly higher rates of disease progression and development of metastases were reported in the active monitoring group compared with either immediate surgery or radiotherapy [13]. As illustrated by the findings of ProtecT, as well as other trials, a key challenge in managing over-treatment is identifying and distinguishing between men with tumors that are likely to progress to clinically significant disease and those whose disease is likely to remain indolent for the remainder of their lifetime. Prostate cancer is a heterogeneous disease and current staging methods such as Gleason grade, whilst a powerful prognostic indicator, are associated with limitations. In particular, there may be inter-observer variability in determining Gleason grade and tumor tissue may be heterogeneous and may be multifocal in origin, which biopsy may not detect [14, 15]. Additionally, reported sensitivity rates for transrectal ultrasound-guided biopsy range from 49 to 87%, whilst specificity ranges from 38 to 93% [16]. There is currently a significant research effort directed towards characterizing and better understanding biomarkers that can aid prognosis and predict risk of recurrence and several genomic profiling tools have recently been developed, although the use of such tools in clinical practice is not currently widespread. Future research efforts in this area may help to identify to better distinguish between those patients whose disease is likely to progress versus those whose disease is likely to remain indolent or not clinically significant and therefore help to reduce over-treatment.

The high incidence of men with prostate cancer requiring treatment, as well as those with elevated PSA levels or low risk prostate cancer undergoing watchful waiting or active surveillance, means that the economic burden of prostate cancer is also substantial. In the UK alone, total annual costs for treatment in the first year following diagnosis have been estimated at approximately EUR 117 million and in France and Germany this figure is two- to three-fold higher [17]. Additionally, in the UK the active surveillance approach advocates repeat biopsy at 12 months after an observed elevation in PSA levels as well as regular follow up PSA tests and DREs. This approach is therefore also associated with notable medical resource utilization, although direct medical costs for these patients are likely to be substantially lower than for those patients undergoing treatment. In some settings, MRI is being increasingly used in to help guide biopsies and can improve the detection of clinically significant tumors relative to standard biopsy approaches alone, as well as providing the potential to exclude clinically insignificant tumors and reduce the requirement for subsequent biopsies [18]. Indeed, findings from the recently published PRECISION trial showed that a strategy of MRI prior to MRI-targeted biopsy (if required) was superior to standard transrectal ultrasonography-guided biopsy in terms of detecting clinically significant cancer [19]. MRI can also be used following negative transrectal ultrasound biopsy in high risk patients where it can be used to guide subsequent biopsies [18, 20], MRI also has an emerging role in active surveillance and detection of local recurrence following treatment, all of which have implications in terms of medical resource utilization [20].

Several factors contribute to the overall burden of disease of prostate cancer, these include the incidence and mortality rates as well as direct and indirect costs, the humanistic burden associated with the impact of a diagnosis of prostate cancer on quality of life as well as the burden placed on the caregivers of men with prostate cancer. To characterize the burden of prostate cancer in Europe and North America (using the specific examples of Germany, France, the United Kingdom and Canada) in terms of incidence, cost and impact on quality of life of prostate cancer, a literature review of recently published articles was undertaken.

## Methods

Literature search strategies were designed to capture recently published data relating to the clinical, economic and humanistic burden of prostate cancer in France, Germany, the United Kingdom and Canada. Search strategies were designed using high level Medical Subject Heading (MeSH) terms supplemented with free text terms with the search syntax adapted as required for use in the PubMed, EMBASE and Cochrane Library database (full details of the search strategies used are available in the Additional file 1). Searches were limited to full text articles published in English since 2006. The scope of the search was limited to articles conducted in populations in France, Germany, the United Kingdom or Canada, or presenting data according to country-based subgroups including either France, Germany, the United Kingdom or Canada. For inclusion, epidemiologic studies were also required to report incidence and/or mortality rates for the overall or general population, consequently, studies presenting data only within or according to population subgroups (e.g. only reporting data from one age group, ethnic group or socioeconomic group) were excluded, secondary sources of data were also excluded. All searches were performed on 7 April, 2016.

## Results

### Literature searches

Literature searches across the PubMed, EMBASE and Cochrane Library identified a total of 2966 articles. After removal of duplicates a total of 2772 unique articles were screened. An initial first round screen of title and abstracts was performed to identify potentially relevant articles for full text screening. A total of 163 articles were identified for full text screening and a total of 49 articles were included in the final review.

### Incidence, mortality and survival

A total of 16 articles reported the incidence of prostate cancer either in the overall population or in a representative sample of the overall population in France, Germany, the UK or Canada [21–36]. Although the searches required articles to be published from 2006 onwards, several articles published incidence data from before this cut off point. In both France and Germany, annual age-standardized incidence figures from 2005 onwards were consistently in the region of approximately 100–130 per 100,000 (Table 1). In terms of absolute numbers of cases, in both France and Germany, more than 50,000 incident cases were reported annually from 2004 onwards.

**Table 1:** Incidence of prostate cancer in France, Germany, the United Kingdom and Canada

Study (year)	Year of data	Age standardized incidence per 100,000	Incident number of cases, n
France			
Binder-Foucard et al. (2014) [21]	1980	24.8	―
	2005	127.1	―
	2012	―	53,465 (95% CI: 46,840 to 60,090)
Crocetti et al. (2013) [22]	1998–2002	122	183,136
Bray et al. (2010) [23]	1988–2002	115.0	―
Belot et al. (2008) [24]	1980	23	10,756
	1985	33	14,190
	1990	42.2	18,979
	1995	56.5	26,760
	2000	80.4	39,636
	2005	121.2	62,245
Germany			
Haberland et al. (2010) [25]	2004	112.0	58,574
	2015	―	70,904 (projected)
	2020	―	76,034 (projected)
Dorr et al. (2015) [26]	1990–1992	55.2	―
	2008–2010	106.0	―
Becker et al. (2007) [27]	2002	147.9 (crude)	―
Rohde et al. (2009) [28]	2001–2002	101.9	―
	2002–2003	125.6	―
	2004–2005	101.9	―
Bray et al. (2010) [23]	1975–2002	109.8	―
United Kingdom			
Greenberg et al. (2013) [29]	2000–2005 (total cases)	365.11	―
	2000–2005 (metastasis)	58.2	―
	2006–2010 (total cases)	416.73	―
	2006–2010 (metastasis)	51.13	―
Mistry et al. (2011) [30]	1984	40.5	11,714
	2007	97.2	36,083
	2030	104.8	61,089
Pashayan et al. (2006) [31]	1971	32	―
	2000	89	―
Shafique et al. (2012) [32]	1991	44	―
	2007	75	―
Feletto et al. (2015) [33]	2011	107.4	―
Westlake (2009) [34]	2004–2006	98.3	―
Bray et al. (2010) [23] (England and wales)	1980–2006	87.5	―
Canada			―
Kachuri et al. (2013) [35]	1970	53.8	―
	2007	124.7	―
Feletto et al. (2015) [33]	2007	133	―
Louchini et al. (2008) [36]	1988–2004	67.1 (66.5–67.7)	―

For the UK, the range of reported incidence figures from 2007 onwards showed greater variation from a low of 75 per 100,000 in 2007 in a study conducted in the West of Scotland using data from the Scottish Cancer Registry [32] to a high of 417 per 100,000 in a study utilizing data from the Anglican Cancer Network [29]. Only one UK-based study presented the absolute number of incident cases, here Mistry et al. reported that in 2007 there were > 36,000 incident cases of prostate cancer as well as projecting that by 2030 this figure will increase to > 61,000 [30].

One of the studies included in the review was a European-wide study of prostate cancer incidence and mortality over the past two decades [23]. This study showed a geographic gradient in prostate cancer incidence with the highest incidence rates reported in Northern Europe and the lowest in Eastern Europe. Analysis of incidence trends over the last two decades showed annual increases in prostate cancer of between 3 and 7% in most countries, which concurs with the findings of other studies included in the current review. However, in the final years of the multinational study by Bray et al. decreases in incidence were reported in several high incidence countries.

Only two studies reported age-standardized incidence in Canada from 2007 onwards, both of which were broadly consistent with incidence figures ranging from 125 to 133 per 100,000 in 2007 [33, 35]. Additionally, one of the Canada-based studies examined incidence rates over time, reporting that in 1970 age-standardized prostate cancer incidence was 54 per 100,000, showing that as in Western European settings the incidence of prostate cancer in Canada has increased steadily over time [35].

A total of 11 studies reported mortality rates in men with prostate cancer in France, Germany the UK and Canada [21, 23–25, 27, 28, 33–37]. The lowest mortality rates were reported in French studies, where the mortality rate for post-2005 studies ranged from 10 to 14 per 100,000 population [21, 24]. Two French studies also reported the absolute number of deaths attributable to prostate cancer; in France from 1995 onwards there were approximately 9000 prostate cancer deaths per year [21, 24], whilst in Germany in 2004 approximately 11,000 men died due to prostate cancer [25]. In the UK, Germany and Canada mortality rates from 2003 were broadly similar across all three settings ranging from 17 to 28 per 100,000 (Table 2). Studies that examined mortality rates over time consistently reported declining mortality over time [21, 24, 28, 35]. In particular, in Germany mortality rates decreased by 20% over the period 1999–2005 [28].

**Table 2:** Prostate cancer-related mortality in France, Germany, the United Kingdom and Canada

Study (year)	Year of data	Age standardized mortality per 100,000	Number of deaths, n
France			
Binder-Foucard et al. (2014) [21]	1980	16.3	―
	2012	10.2	8876
Bray et al. (2010) [23]	1975–2007	24.9	―
Belot et al. (2008) [24]	1980	16.9	7001
	1985	17.8	8090
	1990	17.7	8875
	1995	16.7	9279
	2000	15.3	9295
	2005	13.5	9202
Germany			
Haberland et al. (2010) [25]	2004	22.2	11,135
Becker et al. (2007) [27]	2002	28.3 (crude)	―
Rohde et al. (2009) [28]	2001–2002	29.3	―
	2002–2003	28.9	―
	2004–2005	27.9	―
Bray et al. (2010) [23]	1975–2006	23.3	―
United Kingdom			
Feletto et al. (2015) [33]	2010	23.8	―
Westlake (2009) [34]	2004–2006	25.7	―
Bray et al. (2010) [23] (England and Wales)	1975–2007	26.8	―
Marshall et al. (2016) [37]	2003–2007	25.7	―
Canada			
Kachuri et al. (2013) [35]	1970	25.4	―
	2007	20.4	―
Feletto et al. (2015) [33]	2011	16.7	―
Louchini et al. (2008) [36]	1988–2004	16.8(16.5–17.1)	―

In addition to mortality figures, a total of six studies reported 5-year relative survival rates. One study was multinational [38] and the remaining five were conducted in Germany [26, 39–42]. In Germany, 5-year relative survival rates ranged from 81%, reported over the period 1995–2003 [42] to 95%, reported over the period 2005–2010 [26]. The multinational study by Trama et al. reported a 5-year survival rate over the period 2000–2007 of 89% for men in both France and Germany; however, for the UK, the corresponding figure was lower at 81% [38]. Additionally, UK-based evidence from Cancer Research UK, demonstrate the strength of the relationship between stage at diagnosis and 5-year relative survival. For men diagnosed with stage I prostate cancer, 5-year relative survival in the UK is 112% (implying that these men live longer than the general population), which decreases to 93% for men diagnosed with stage III disease and 30% for men diagnosed with stage IV disease [43].

A multinational study by Bray et al. also noted declining mortality rates were seen in a total of 13 countries, which were primarily high-income countries in Western Europe. The decreases in mortality that were seen over time in several studies have been attributed to increased uptake of opportunistic PSA screening from the early 1990s onwards leading to detection of prostate cancer at earlier stages as well as changes in treatment uptake and continual improvements in treatment modalities. However, the evidence for the reduced mortality due to increased PSA screening is not conclusive. For example, one study from the Munich Cancer Registry showed that treatment modalities have changed over time and that use of radical prostatectomy increased from approximately 20 to 50% over the last two decades, whilst the use of hormone therapy has decreased over this period [26]. A “stage shift” towards earlier detection of tumors was also evident in the UK; Greenberg et al. reported a significant increase in overall prostate cancer incidence, but a significant decrease in the incidence of metastatic prostate cancer in the period 2006–2010 compared with 2000–2005 [29]. The existence of a stage shift is however not universally supported as other studies including a UK-based study by Shafique et al. reported no evidence of a stage shift [32].

### Economic burden of prostate cancer

The consensus among studies that investigated temporal trends in prostate cancer incidence is one of increasing incidence over time. This, together with high rates of over-treatment in many settings mean that the economic burden associated with the treatment and monitoring of men is both substantial and growing.

Only one study identified in the literature review examined the overall burden of prostate cancer on a national level [17], however, nine other study reported direct or indirect costs on a per patient level [44–52]. Fourcade et al. examined per patient and total costs for men with prostate cancer in the first year after diagnosis in five European countries (France, Germany, Italy, Spain and the UK) [17]. In the countries of interest, the total overall burden in the first year after diagnosis was highest in France at EUR 385 million, followed by Germany at EUR 244 million and then the UK at EUR 117 million per year (2006 EUR). Further, another study (identified by supplementary hand-searches) reported that in the EU the overall cost (including direct and indirect costs) of prostate cancer in 2009 was EUR 8.43 billion, which constitutes 7% of the overall economic burden of cancer in the EU and of which, EUR 5.43 billion were healthcare costs, EUR 0.73 billion was due to lost productivity and EUR 1.88 billion was attributable to costs associated with informal care [53]. The study by Fourcade et al. also reported per patient costs (excluding follow-up and adverse event costs), which were highest in France at EUR 5851 per patient, followed by Germany at EUR 3698 then the UK at EUR 3682 per patient. In a similar study from Canada, De Oliveria reported that the total healthcare costs in the first 12 months after diagnosis in men aged ≥45 years were CAD 15,170 per patient [47].

Two cost studies included in the review examined direct costs according to cancer stage and/or treatment [44, 50]. In the first of these, Molinier et al. report overall costs of EUR 12,731 per patient (2008 EUR), but when broken down according to stage, in France, patients with regional prostate cancer had the highest total costs at EUR 16,608 per patient, whilst those with metastatic disease had the lowest overall costs at EUR 9994 per patient [44]. Additionally, in terms of costs for specific treatment modalities, Molinier et al. also reported that external beam radiotherapy was consistently associated with the highest direct costs, followed by radical prostatectomy then androgen deprivation therapy. Notably, costs associated with a watchful waiting approach ranged from EUR 4730–9355. Similarly, in a Canadian study direct medical costs (per 100 days in health state) were highest for patients with advanced disease (Table 3).

**Table 3:** Per patient cost of prostate cancer in France, Germany, United Kingdom and Canada

Study (year)	Description of cost	Cost year	Currency	Mean cost
France				
Fourcade et al. (2010) [17]	Total direct cost of treatment in 12 months after diagnosis, excluding follow up and adverse event costs, for all diagnosed patients	2006	EUR	5851
Molinier et al. (2011) [44]	Mean total cost (all prostate cancer)	2008	EUR	12,731
	Mean total cost localized	2008	EUR	12,259
	Mean total cost regional	2008	EUR	16,608
	Mean total cost metastatic	2008	EUR	9994
Germany				
Fourcade et al. (2010) [17]	Total direct cost of treatment in 12 months after diagnosis, excluding follow up and AE costs, for all diagnosed patients	2006	EUR	3698
UK				
Hall et al. (2015) [45]	Cumulative hospital-based costs of care over 15 months following initial diagnosis in patients treated with curative intent	2011/12	GBP	3722 (95% CI, 3263–4208)
Fourcade et al. (2010) [17]	Total direct cost of treatment in 12 months after diagnosis, excluding follow up and AE costs, for all diagnosed patients	2006	EUR	3682
Marti et al. (2016) [46]	Total societal (and component costs) in prostate cancer survivors treated with curative intent over 12–15 months post-diagnosis period			
	Total societal cost (per month)	2012	GBP	117.9 (95% CI: 68.9–167.0)
	NHS costs (per month)	2012	GBP	92.1 (49.3–134.9)
	Patient OOP costs (per month)	2012	GBP	7.2 (2.3–12.0)
	Informal care costs (per month)	2012	GBP	16.4 (−5.5–38.3)
Canada				
De Oliveira et al. (2013) [47]	Total healthcare costs in 12 months after diagnosis in patients aged ≥45 years	2009^a^	CAD	15,170
Krahn et al. (2010) [48]	Direct medical costs for men diagnosed 1995–2002 according to phase	2004	CAD	
	Phase Ib (costs incurred in 6 months prior to prostate cancer diagnosis)	2004	CAD	1904
	Phase IIb (costs incurred in first 12 months after prostate cancer diagnosis)	2004	CAD	12,005
	Phase IIIb (“continued care” phase, costs per 100 days)	2004	CAD	1495
	Phase IVb (pre-terminal care in 18 to 6 months before death)	2004	CAD	20,543
	Phase Vb (terminal care, 6 months before death)	2004	CAD	28,834
De Oliveira et al. (2014) [49]	Patient time costs per year (all)	2006	CAD	838 (442–1233)
	Out of pocket costs per year (all)	2006	CAD	200 (109–290)
Krahn et al. (2014) [50]	Early health states: non-treated, non-metastatic^a^	2008	CAD	3440
	Early health states: radiation therapy^a^	2008	CAD	2160
	Early health states: radical prostatectomy^a^	2008	CAD	4676
	Early health states: hormone therapy^a^	2008	CAD	3357
	Middle health states: post-radiation therapy^a^	2008	CAD	1556
	Middle health states: post-radical prostatectomy^a^	2008	CAD	732
	Middle health states: recurrence/progression^a^	2008	CAD	1919
	Late health states: hormone therapy refractory^a^	2008	CAD	4503
	Late health states: metastatic^a^	2008	CAD	4062
	Late health states: metastatic refractory^a^	2008	CAD	6398
	Late health states: final^a^	2008	CAD	13,739
Sanyal et al. (2016) [51]	Total overall cost (overall cohort) at:			
	5 years	2014	CAD	18,503 (17,851–19,185)
	10 years	2014	CAD	28,032 (26,129–29,973)
	15 years	2014	CAD	39,143 (36,606–41,821)
Dragomir et al. (2014) [52]	Active surveillance; cost first 5 years	2012	CAD	6200 (6083 to 6317)
	Immediate treatment; cost first 5 years	2012	CAD	13,735 (13,615 to 13,855)

^a^Cost per 100 days

The majority of cost studies identified reported direct or total costs, with only two studies reporting indirect costs in terms of patient time costs and also patient out-of-pocket costs. In one Canadian study mean (95% CI) annual patient time costs were CAD 838 (442–1233) (2006 CAD) and annual patient out of pocket costs were CAD 200 (109–290) [49]. However, both patient time and out-of-pocket costs were influenced by several parameters, including age. In particular, men aged ≤60 years had higher patient time and out-of-pocket costs compared with those aged > 60 years, with the difference in out-of-pocket costs being statistically significant (*p* = 0.03) [49]. In the UK, annual mean (95% CI) out-of-pocket costs were GBP 86 (28–144) and mean annual costs for informal care were GBP 197 (− 66–460) [46]. The Canadian study also examined patient time and out-of-pocket costs according to urinary function, bowel function and sexual function according to the Prostate Cancer Index (PCI) score. Patients with the lowest scores for urinary function (score = 0–25) had mean total (patient time and out-of-pocket) costs of CAD 4186 per year, which was almost ten-fold higher than for patients with the highest (75–100) PCI urinary function scores (CAD 444 per year). Similarly, total patient time and out-of-pocket costs for patients with the lowest PCI sexual function scores were almost two-fold higher than those with the highest scores (CAD 1401 versus CAD 728) [49].

### Quality of life in patients with prostate cancer

There are a large number of studies in the literature that either qualitatively or quantitatively assess the impact of prostate cancer on quality of life (QoL), however, the scope of the current literature review was limited to studies reporting health state utility values for men with prostate cancer. In total, nine studies were identified that examined the health status of men with prostate cancer in either France, Germany, the UK or Canada [45, 54–61]. Several different instruments were used in these studies to directly or indirectly assess QoL in men with prostate cancer including the EQ-5D, European Organization for Research and Treatment of Cancer 8-dimensional utility index (EORTC-8D), patient-oriented prostate utility scale (PORPUS-U), Health Utilities Index 2 and 3 (HUI-2; HUI-3) and the Quality of Well Being scale (QWB). Utility values were also reported for several different health states including overall men at risk for prostate cancer, overall populations of men with prostate cancer, men with localized disease and those with metastatic disease as well as pre- and post-treatment utility values. Heterogeneity in the literature in terms of the exact definitions of health states used, treatment modalities and the time period at which QoL was assessed complicates the comparison of QoL between studies. However, the literature consistently showed that QoL is reduced in men with prostate cancer relative to the general population, although the magnitude of impairment was influenced by disease stage, treatment and presence of complications in terms of urinary function, bowel function and sexual function (Table 4). In particular, men with who reported moderate or big problems with bowel function had a mean (SD) utility value (EQ-5D) of 0.653 (0.195) compared with 0.862 (0.166) for those with no problems with bowel function [56]. Other studies conducted in the general population confirm that complications of treatment, including erectile dysfunction, bowel dysfunction and urinary incontinence can have a substantial detrimental impact on QoL. For example, in one multinational study of QoL in men with erectile dysfunction, and their partners, mean utility values, elicited using standard gamble, ranged from 0.40–0.49 [62]. Similarly, QoL is also reduced relative to the general population in people with urinary incontinence [63].

**Table 4:** Quality of life in men with prostate cancer

Study (year)	Health state	Mean (SD or 95% CI) utility value					
		EQ-5D	EORTC-8D	PORPUS-U	HUI-2	HUI-3	QWB
France							
Sullivan et al. (2007) [54]	Metastatic hormone refractory prostate cancer, baseline	0.669					
Germany							
Sullivan et al. (2007) [54]	Metastatic hormone refractory prostate cancer, baseline	0.527					
UK							
Sullivan et al. (2007) [54]	Metastatic hormone refractory prostate cancer, baseline	0.715					
Hall et al. (2015) [45]	Baseline, at diagnosis	0.838					
	9 months post-diagnosis	0.868					
	15 months post-diagnosis	0.868					
Lloyd et al. (2015) [55]	MCRPC asymptomatic/mildly symptomatic before chemotherapy	0.830 (0.126)	0.856 (0.089)				
	MCRPC symptomatic before chemotherapy	0.625 (0.173)	0.697 (0.118)				
	MCRPC currently receiving chemotherapy	0.692 (0.219)	0.750 (0.117)				
	MCRPC post chemotherapy	0.700 (0.183)	0.753 (0.133)				
Watson et al. (2016) [56]	Diagnosed with prostate cancer in previous 9–24 months (overall)	0.852 (0.173)					
	No moderate/big problem with urine function	0.868 (0.160)					
	No moderate/big problem with bowel function	0.862 (0.166)					
	No moderate/big problem with sexual function	0.861 (0.176)					
	Moderate/big problem with urine function	0.773 (0.222)					
	Moderate/big problem with bowel function	0.653 (0.195)					
	Moderate/big problem with sexual function	0.838 (0.170)					
Canada							
Krahn et al. (2013) [57]	Prostate cancer survivors (overall)			0.91 (0.08)	0.85 (0.15)	0.78 (0.24)	
Krahn et al. (2007) [58]	Localized prostate cancer receiving radical prostatectomy, radiation, or hormonal therapy	0.84			0.87	0.81	0.61
	Metastatic disease	0.84			0.86	0.82	0.63
	Stable patients	0.85			0.86	0.81	0.63
Ku et al. (2009) [59]	Patients undergoing radical prostatectomy, baseline (pre-surgery)			0.94 (0.93–0.95)			
	Patients undergoing radical prostatectomy, 0–3 months post surgery			0.81 (0.79–0.82)			
	Patients undergoing radical prostatectomy, 3–9 months post surgery			0.87 (0.86–0.89)			
	Patients undergoing radical prostatectomy, 9–18 months post surgery			0.89 (0.87–0.90)			
	Patients undergoing radical prostatectomy, 18–30 months post surgery			0.90 (0.88–0.91)			
Sullivan et al. (2007) [54]	Metastatic hormone refractory prostate cancer, baseline	0.750					
Gries et al. (2016) [60]	Men at risk for prostate cancer				0.83 (0.168)	0.77 (0.238)	
	Men with prostate cancer				0.83 (0–124)	0.75 (0.260)	
	General population				0.87 (0.136)	0.84 (0.178)	
Bremner et al. (2007) [61]	Men with prostate cancer (overall population)					0.80 (0.19)	0.65 (0.13)

*MCRPC* metastatic hormone resistant prostate cancer

One multinational study presented utility values (using the EQ-5D) for men with metastatic hormone refractory prostate cancer reported health state utility values ranging from 0.527 for men in Germany to 0.750 for men in Canada [54]. Interestingly, these values are notably lower than those reported by Krahn et al. for Canadian men with metastatic disease, who had a mean utility value (using the EQ-5D) of 0.84, which was the same as that for men with localized prostate cancer who were receiving treatment (radical prostatectomy, radiotherapy or hormonal therapy) [58]. Lloyd et al. also assessed QoL in UK-based men with metastatic castration-resistant prostate cancer using both the EQ-5D and EORTC-8D questionnaires. Using the EQ-5D Lloyd et al. report a mean (SD) utility value of 0.830 (0.126) for men who were asymptomatic or mildly symptomatic before chemotherapy, whilst for men who were symptomatic this value was substantially lower at 0.625 (0.173) [55]. However, the same study reported higher values for the same health states when utility values were elicited using the EORTC-8D questionnaire. For example, the mean (SD) utility value for men with metastatic castration resistant prostate cancer currently receiving chemotherapy was 0.692 (0.219) using the EQ-5D and 0.750 (0.117) using the EORTC-8D [55]. A Canadian study by Krahn et al. (2007) also examined QoL using several different instruments [58]. Utility values were broadly similar between the HUI-2, HUI-3 and EQ-5D, however utilities elicited using the QWB were notably lower. Krahn et al. also noted that disease-specific instruments (e.g. PORPUS), were more sensitive and had better internal responsiveness than generic QoL instruments. Lower utility scores with the QWB relative to the HUI-3 were also reported in another Canadian study by Bremner et al., who reported a mean (SD) utility for men with prostate cancer of 0.80 (0.19) using the HUI-3 but 0.65 (0.13) using the QWB [61].

Overall, literature on QoL shows that prostate cancer is associated with a decrement in QoL, but that treatment and treatment-related side effects are also associated with deficits in QoL.

## Discussion

### Incidence, mortality and survival

Evidence from epidemiologic studies revealed a consistent trend for a continuing increase in the incidence of prostate cancer across all four settings included in the review. The increases in incidence, particularly of low grade tumors, reported in many settings have been partly attributed to the introduction of PSA screening in the early 1990s as well as increasing life expectancy. However, the etiology of prostate cancer is unclear so other factors may be contributing to incidence trends. Age, ethnicity and family history are known to be the major risk factors for prostate cancer; the role of diet and other risk factors are thought to be only minor influencing factors in overall risk and, moreover, studies on dietary risk factors have yielded inconsistent results. Additionally, between study differences in incidence rates may also be due in part to differences in population groups in different studies and also the methods and/or quality of data collection used.

Evidence from included studies also showed that relative survival rates have improved over time, although the 5-year relative survival rate in the UK was found to be lower than in both France and Germany. Improved relative survival in the post- versus pre-PSA screening era has also been reported in other European countries, including one recently published study from Finland where improved 5-year relative survival was reported for both localized and metastasized prostate cancer [64, 65]. In these studies improved survival outcomes were at least partly attributed to earlier diagnosis owing to PSA screening. Although it was also noted that PSA screening can result in lead-time bias in survival estimates, where lead-time is “*the time by which PSA screening advances prostate cancer diagnosis*” [65].

Notably, in their European-wide study, Bray et al. noted a lack of correlation between incidence and mortality rates, particularly in the later years of their study, which they suggest was due to the detection and over-diagnosis of indolent tumors, which was in turn likely attributable to increasing uptake of PSA screening [23]. Similar findings have been reported in a meta-analysis of population screening studies, which showed that whilst PSA screening has led to a significant increase in prostate cancer detection as well as a significant grade shift towards lower grade tumors, it has not significantly influenced mortality rates [66].

A key limitation of this review is that although the overarching goal was to characterize the burden of prostate cancer in contemporary clinical practice a number of included articles, although published recently, did not contain recent data. The scope of the literature searches was limited to articles published after 2006. However, a substantial proportion of the articles meeting this cut-off threshold reported data from earlier, often considerably earlier, time periods. Therefore, currently available data on incidence and prostate cancer-specific mortality may not accurately reflect the current situation in contemporary clinical practice. A further limitation is that in terms of 5-year relative survival data included in the review, the most recent data are from 2010, which again may not accurately reflect the current situation in routine clinical practice. Allied to this, in the area of oncology long-term follow-up (typically 15–20 years) is required to fully investigate and elucidate the underlying mechanisms for changes in mortality rates. Only a small number of studies included in the review were conducted over time horizons of > 15 years.

Evidence from several studies showed that in the UK in particular, the incidence of prostate cancer is influenced by ethnic group and socioeconomic status. Specifically, UK-based men of African, or African-Caribbean descent have been consistently reported to have higher incidence rates whilst those of South Asian descent had consistently lower incidence rates of prostate cancer relative to those of European descent [67–69] In particular, the rate ratio for Black African and Black Caribbean men in the UK ranged from 2.4–3.1 compared with white males, whilst for South Asian males the corresponding range was 0.3–0.7 [67, 68]. However, Chingewundoh et al. reported no notable differences between ethnic groups in terms of clinical presentation of prostate cancer, with no significant differences reported in mean PSA score, stage or Gleason score at presentation [69]. Differences in incidence according to ethnic group were also reported in Canada, with Aboriginal men in Quebec having a substantially lower age-standardized incidence of prostate cancer compared with the general population (47 [38–57] per 100,000 versus 67 [67, 68] per 100,000) [36]. Additionally, evidence from a UK-based study from the West of Scotland showed that from 1998 onwards a deprivation gap appeared in the incidence of low grade, but not high grade, prostate cancer, with men in the most deprived socioeconomic group having a 37% lower incidence of low grade prostate cancer compared with men in the most affluent group [32]. However, the authors note that the reason for this deprivation gap is unclear, stating that possible explanations include a difference in causal factors and competing mortality, as incidence is strongly related to age although the difference is unlikely to have been due to PSA screening as the overall increase in incidence was not accompanied by a grade shift towards lower grade disease.

Although not included in the current review (as data were reported in graphical rather than numerical format) Collin et al. compared trends in prostate cancer mortality in the UK and the US over the period 1975–2004 [70]. In both settings, mortality rate declined from 1994 onwards, but the rate of decline in the US was four-fold greater than in the UK. This difference was attributed to a combination of several factors including much higher uptake rates of PSA screening in the US as well as a more aggressive approach towards management of early stage treatment. However, it should also be noted that there have been considerable advances in the treatment of prostate cancer since 2004 [71], which may have influenced mortality rates in both the UK and US.

### Economic burden of prostate cancer

Overall, the findings from studies included in the current review suggest that the economic burden associated with the treatment and monitoring of men with prostate cancer is both substantial and increasing over time. Factors contributing to the increasing economic burden include increasing incidence, advances in treatment and advances in diagnostic and monitoring technologies such as the increased use of MRI and the introduction of genomic profiling tools. However, it should be noted that some of the cost data included in the current review may not adequately capture the direct costs, or savings, associated with the most recent advances in the treatment of prostate cancer. For example, increased use of robot-assisted radical prostatectomy is associated with increased surgical costs relative to open or laparoscopic radical prostatectomy [72]. Additionally, increasing use of advances such as prostate-specific membrane antigen based PET or CT imaging and 3 T MRI in the diagnosis of prostate cancer are also likely to have influenced the overall economic burden of disease.

One aspect of economic burden that was not well characterized in the literature was costs associated with treatment-related complications that some patients experience such as incontinence, bowel dysfunction and erectile dysfunction. However, other studies, not included in the review have examined costs in patients with these conditions. For example, one publication (excluded from the current review as is was not published in English) estimated that in Germany the direct and indirect costs associated with urinary incontinence following prostatectomy are approximately EUR 71.8 million [73]. Similarly, figures from the UK estimate that on a per patient level, the annual cost of erectile dysfunction is GBP 335 [74].

There is a general paucity of data relating to indirect costs for patients with prostate cancer, and intangible aspects such as the burden placed on informal caregivers is poorly characterized. The literature searches did not identify any studies relating to caregiver burden in the settings of interest (UK, Canada, Germany and France). However, two US-based studies have quantified caregiver burden in the partners of patients with prostate cancer. In one study Li et al. reported that in the year following diagnosis, mean partner working hours of men with localized prostate cancer (who were not receiving treatment other than hormone therapy) decreased from 14.0 to 10.9 h per week [75]. Additionally, a mean (SD) of 1.3 (3.3) hours per week was spent on providing informal care and 1.5 (3.7) hours/week were spent performing household tasks that would otherwise have been performed by the patient, leading to a total of 276 h per year of lost productivity, informal care and additional household tasks. In economic terms the authors reported that caregiving and lost productivity translated to an economic burden of USD 6063 per patient. A second US-based study estimated caregiver burden to be substantially higher in the first 2 years following diagnosis and a mean (SD) of 9.1 (8.8) hours per day was spent on providing informal care for a partner with prostate cancer. Using the human capital approach the mean (95% CI) economic value of informal care over this time period was USD 44,885 (35,389–54,381) (2006 USD) [76]. Additionally, another US-based study showed that in addition to the burden associated with informal care, the partners of men with prostate cancer had reduced QoL, with partner QoL being negatively influenced in particular by impotence, incontinence and the presence of metastatic disease [77, 78].

A key caveat of high levels of PSA screening is high levels of over-diagnosis and over-treatment. Over-treatment compounds not only the clinical burden of disease but side effects of treatment including impotence, incontinence and impaired bowel function that can compromise quality of life for both the patient and patient’s partner. The economic burden of over-treatment is also considerable with the annual burden of prostate cancer in the UK, France and Germany ranging from EUR 117–385 million.

However, the burden of over treatment could be reduced by identifying and treating only those tumors that are likely to progress to clinically significant disease. Several genomic profiling tools that assess the expression of predictive and prognostic biomarkers in prostate cancer are now available. These include Decipher™, Oncotype DX®, and Prolaris®, the use of which may assist physicians with decision-making in terms of the risk/benefit profile of initiating treatment in men with low grade disease as well as potentially reducing the requirement for repeat biopsies in men with low grade disease assigned to watchful waiting or active surveillance. The use of such tools can enable a more personalized approach to treatment and the quantitative assessment of RNA levels in some genomic profiling tools also enables an assessment of the influence of epigenetics on gene expression in prostate tumors. However, there are substantial differences between the different tools in terms of the genes assayed and number of genes assays (e.g. Oncotype DX® Genomic Prostate Score™ incorporates multiple biologic pathways predictive of prostate cancer aggressiveness, whereas the 4 K score test exclusively examines plasma levels of four kallikrein proteins), the prognostic and/or predictive information provided, the quality and reproducibility of the test (e.g. Oncotype DX® is performed in central laboratory using standard operating procedures) and the weight of supporting clinical evidence available for each test. Additionally, many of these tools are relatively new and as such are not yet routinely used in routine practice, and cost may represent a barrier to uptake in some settings, but the development and increased use of such genomic profiling tools over the coming years may help to reduce rates of over-treatment.

### Quality of life

The QoL studies included in the review consistently showed that QoL was reduced not only in men with prostate cancer but also in the partners of men with prostate cancer. Additionally, treatments such as radical prostatectomy, whilst curative for many patients, can lead to lifelong problems that compromise QoL including urinary incontinence and impotence. The burden on the caregivers, who are often partners, of men with prostate cancer is often overlooked in the literature, but the small amount of research on this area that does exist has shown that partners spend a considerable amount of time providing informal care as well as reducing their working hours in order to do this.

## Conclusions

Prostate cancer is the most common cancer in men in Europe and North America, with incidence rates projected to continue to increase over the coming years and whilst advances in treatment have led to substantial improvements in mortality rates, the long-term burden of disease could be reduced by development and increased use of genomic profiling tools, which may help to reduce the over-treatment of indolent tumors.

## Additional file

Additional file 1: Literature searches: Details of search strategies used in the literature review and number of hits returned. (DOCX 21 kb)
