# Supplementary material for: Literature review of the burden of prostate cancer in Germany, France, the United Kingdom and Canada
Source: BMC Urol. 2019 Mar 18;19:19. doi: 10.1186/s12894-019-0448-6 (PMC6421711; doi:10.1186/s12894-019-0448-6)
Supplement: Supplementary file 1 — Literature searches: Details of search strategies used in the literature review and number of hits returned. (DOCX 21 kb) [file 12894_2019_448_MOESM1_ESM.docx]

# Additional file 1

Table 1 PubMed search strategy

| **Line #** | **Search Terms** | **Hits** |
| --- | --- | --- |
| #1 | prostatic neoplasms[majr] OR cancer, prostate[majr] OR ((prostate[tiab] OR prostatic[tiab]) AND cancer[tiab]) | 123,042 |
| #2 | epidemiology[mesh] OR prevalence[mesh] OR incidence[mesh] OR population[mesh] OR patterns, physician's practice[mesh] OR prevalence[tiab] OR incidence[tiab] OR epidemiology[tiab] | 1,490,627 |
| #3 | (“Costs and cost analysis”[MeSH] OR “cost effectiveness”[tiab] OR “cost utility”[tiab] OR cost minimi*[tiab] OR “cost consequence”[tiab] OR budget*[tiab] OR economic model*[tiab] OR “Markov chains”[MeSH] OR markov*[tiab] OR “Monte carlo method”[MeSH] OR monte carlo[tiab] OR”Models, economic”[MeSH] OR “Models, statistical”[MeSH] OR “Decision Theory”[MeSH] OR decision tree*[tiab] OR decision analy*[tiab] OR decision model*[tiab] OR disease model* OR natural history model*) | 834,708 |
| #4 | (“Quality of Life”[Mesh] OR “Questionnaires”[Mesh] OR “Health Status”[Mesh] OR “Health Status Indicators”[Mesh] OR “Activities of Daily Living”[Mesh] OR “Quality-Adjusted Life Years”[Mesh] OR “Health Surveys”[Mesh] OR “Treatment Outcome”[Mesh] OR “Psychometrics”[Mesh] OR “psychology” [Subheading] OR SF6D[tiab] OR SF 6D[tiab] OR short form 6D[tiab] OR SF-6D[tiab] OR Euroqol[tiab] OR euro qol[tiab] OR eq5D[tiab] OR eq 5D[tiab] OR eq-5D[tiab] OR Utility[tiab] OR disutility[tiab] OR utilities[tiab] OR disutilities[tiab] OR Quality of life[tiab] OR HUI[tiab] or HUI1[tiab] OR HUI2[tiab] OR HUI3[tiab] OR Standard gamble[tiab] OR time trade off[tiab] OR time tradeoff[tiab] OR TTO[tiab]) | 2,199,146 |
| #5 | #1 AND (#2 OR #3 OR #4) | 34,876 |
| #6 | #5 AND "2006/01/01"[PDAT] : "2016/04/07"[PDAT] | 21,500 |
| #7 | #5 NOT  Letter[ptyp] OR Editorial[ptyp] OR Patient Education Handout[ptyp] OR Case reports[ptyp] OR Comment[ptyp] | 20,049 |
| #7 | #6 AND  Canada[MeSH] OR France[Mesh] OR Germany[Mesh] OR Great Britain[Mesh] OR Canada[tiab] OR Canadian[tiab] OR France[tiab] OR French[tiab] OR Germany[tiab] OR German[tiab] OR Great Britain[tiab] OR British[tiab] OR England[tiab] or English[tiab] or United Kingdom[tiab] or UK[tiab] | 1,369 |

Table 2 Cochrane Library search strategy

| **Line #** | **Search Terms** | **Hits** |
| --- | --- | --- |
| #1 | MeSH descriptor: [Prostatic Neoplasms] explode all trees OR "prostate cancer":ti,ab,kw (Word variations have been searched) | 6,473 |
| #2 | MeSH descriptor: [Epidemiology] explode all trees OR MeSH descriptor: [Prevalence] explode all trees OR MeSH descriptor: [Incidence] explode all trees OR epidemiology or prevalence or incidence:ti,ab,kw (Word variations have been searched) | 83,916 |
| #3 | MeSH descriptor: [Costs and Cost Analysis] explode all trees OR MeSH descriptor: [Quality of Life] explode all trees OR MeSH descriptor: [Health Status] explode all trees OR MeSH descriptor: [Health Status Indicators] explode all trees OR MeSH descriptor: [Quality-Adjusted Life Years] explode all trees OR cost of illness:ti,ab,kw (Word variations have been searched) OR cost utility or cost minimization or cost-effectiveness or budget or economic model:ti,ab,kw (Word variations have been searched) OR quality of life or questionnaires or health status or SF6D or EQ5D or Euroqol or disutility or utility or HUI or standard gamble or time trade off or TTO:ti,ab,kw (Word variations have been searched) | 135,395 |
| #4 | #1 AND (#2 OR #3) | 2,160 |
| #5 | #4 AND  MeSH descriptor: [Canada] explode all trees OR MeSH descriptor: [France] explode all trees OR MeSH descriptor: [Germany] explode all trees OR MeSH descriptor: [Great Britain] explode all trees OR France or French or Germany or German or Canada or Canadian or UK or United Kingdom or Great Britain or British or England or English:ti,ab,kw (Word variations have been searched) | 187 |
| #6 | #5 AND  Publication Year from 2006 to 2016 | 141 |

Table 3 EMBASE search strategy

| **Line #** | **Search Terms** | **Hits** |
| --- | --- | --- |
| #1 | 'prostatic neoplasms'/exp OR 'prostatic neoplasms' OR 'prostate cancer'/exp OR 'prostate cancer' OR ('prostate'/exp OR 'prostate' OR 'prostatic' AND ('cancer'/exp OR 'cancer')) | 212,823 |
| #2 | 'prevalence'/exp OR 'prevalence':jt,ab OR 'epidemiology'/exp OR 'epidemiology':jt,ab OR 'incidence'/exp OR 'incidence':jt,ab | 3,044,962 |
| #3 | 'cost analysis'/exp OR 'cost analysis':jt,ab OR 'cost of illness'/exp OR 'cost of illness':jt,ab OR ‘quality of life’/exp OR ‘quality of life’:jt,ab OR ‘health status’/exp OR ‘health status’:jt,ab OR ‘health status indicator’/exp OR ‘health status indicator’:jt,ab OR ‘quality adjusted life year’/exp OR ‘quality adjusted life year’:jt,ab OR ‘health survey’/exp OR ‘health survey’:jt,ab OR utility:jt,ab OR utilities:jt,ab OR disutility:jt,ab OR disutilities:jt,ab | 916,766 |
| #4 | #1 AND (#2 OR #3) | 52,984 |
| #5 | #4 AND  Canada/exp OR 'canada':jt,ab OR 'Canadian':jt,ab OR France/exp OR 'France':jt,ab OR 'French':jt,ab OR Germany/exp OR 'Germany':jt,ab OR 'German':jt,ab OR United Kingdom/exp OR 'United Kingdom':jt,ab OR 'British':jt,ab OR 'English':jt,ab OR 'England':jt,ab OR 'Britian':jt,ab | 13,432 |
| #6 | #5 AND [english]/lim AND [2006-2016]/py AND ([article]/lim OR [article in press]/lim OR [review]/lim) NOT [medline]/lim | 1,461 |
